# Exploring Macrophage-Dependent Wound Regeneration During Mycobacterial Infection in Zebrafish

**DOI:** 10.3389/fimmu.2022.838425

**Published:** 2022-03-24

**Authors:** Candice Bohaud, Matt D. Johansen, Béla Varga, Rafael Contreras-Lopez, Audrey Barthelaix, Claire Hamela, Dora Sapède, Thierry Cloitre, Csilla Gergely, Christian Jorgensen, Laurent Kremer, Farida Djouad

**Affiliations:** ^1^ IRMB, Univ Montpellier, INSERM, Montpellier, France; ^2^ Centre National de la Recherche Scientifique UMR 9004, Institut de Recherche en Infectiologie de Montpellier (IRIM), Université de Montpellier, Montpellier, France; ^3^ Centre for Inflammation, Faculty of Science, Centenary Institute and University of Technology Sydney, Sydney, NSW, Australia; ^4^ L2C, Univ Montpellier, CNRS, Montpellier, France; ^5^ Clinical Immunology and Osteoarticular Diseases Therapeutic Unit, Department of Rheumatology, Lapeyronie University Hospital, Montpellier, France; ^6^ INSERM, IRIM, Montpellier, France

**Keywords:** zebrafish, regeneration, infection, *Mycobacterium marinum*, macrophages sub-types, necrosis

## Abstract

The molecular and cellular mechanisms associated with tissue degradation or regeneration in an infectious context are poorly defined. Herein, we explored the role of macrophages in orchestrating either tissue regeneration or degradation in zebrafish embryos pre-infected with the fish pathogen *Mycobacterium marinum*. Zebrafish were inoculated with different infectious doses of *M. marinum* prior to fin resection. While mild infection accelerated fin regeneration, moderate or severe infection delayed this process by reducing blastemal cell proliferation and impeding tissue morphogenesis. This was correlated with impaired macrophage recruitment at the wound of the larvae receiving high infectious doses. Macrophage activation characterized, in part, by a high expression level of *tnfa* was exacerbated in severely infected fish during the early phase of the regeneration process, leading to macrophage necrosis and their complete absence in the later phase. Our results demonstrate how a mycobacterial infection influences the macrophage response and tissue regenerative processes.

## Introduction

Most mammalian tissues, organs or limbs have only a weak capacity for regeneration. Conversely, some vertebrates, including zebrafish, have acquired the ability to regenerate a tissue or an organ identical to the one lost after a lesion or ablation, in terms of mass, structure and function (1). Therefore, understanding the mechanisms of epimorphic regeneration in vertebrates capable of regenerating their tissues throughout their lives represents an attractive challenge for the future development of innovative therapies in regenerative medicine.

The processes that govern regeneration have been extensively studied in recent years but most were conducted under non-infectious conditions (2, 3). However, there are many examples where lesions inducing a regenerative process occur during infectious diseases. Under these conditions, the presence of pathogenic microorganisms may possibly impact on the regeneration process. Thus, understanding the mechanisms of regeneration in an organism with great regenerative capacities, such as the zebrafish in an infectious environment, is instrumental to allow new discoveries in regenerative medicine with possible applications in patients with infectious diseases.

Macrophages are known to be central players in the regeneration process (4, 5). These hyperplastic cells can adopt various phenotypes in response to different stimuli. Dichotomous characterization of macrophages has led to the discovery of pro- and anti-inflammatory/pro-remodeling or M1- and M2-like macrophage subtypes, respectively, although recognizing the large spectrum of macrophage activation states between this binary classification remains difficult (6, 7).

*In vivo*, live imaging represents a powerful platform for the identification of macrophage phenotypic plasticity and to decipher the kinetics of macrophage subset recruitment and activation during tissue degradation of regeneration following infection. While current murine models are limited in their application to visualize live cellular trafficking for extended periods of time, zebrafish larvae exhibit several advantages such as easy genetic manipulation, optical transparency, and the availability of a wide panel of fluorescent reporter lines to track macrophages (8). Furthermore, the generation of a transgenic zebrafish line allowing the discrimination of pro- and non-inflammatory macrophages during intravital imaging (9) has allowed us to accurately determine the kinetic of recruitment of macrophage subsets during caudal fin regeneration in a pathogen-free context (10). Using this zebrafish transgenic line, we have previously shown that the macrophage subtypes are highly conserved between zebrafish and humans (9). Moreover, zebrafish infection with *Mycobacterium marinum*, a mycobacterial species closely related to *Mycobacterium tuberculosis* and a natural fish pathogen, represents a useful host-pathogen pairing which recapitulates important aspects of tuberculosis and models the human granulomatous immune response to *M. tuberculosis* infection (11–13). The zebrafish *M. marinum* pathosystem has been proven particularly powerful in its application to compare the virulence phenotypes of various *M. marinum* mutants regarding to i) host-pathogen interactions with a special focus on the role of macrophages in the response to infection (12); ii) the underlying mechanisms governing the granulomatous response (11); and iii) novel host-directed therapeutic developments for the treatment of tuberculosis patients (14).

In this study, we addressed whether a pre-established infection with *M. marinum* affects the regeneration process following caudal fin transection in zebrafish. We also inquired whether, depending on the infectious dose inoculated, infection and regeneration may compete for monopolizing the macrophage response at the wound site.

## Results

### Low and High Infectious Doses Respectively Accelerate and Impede Caudal Fin Regeneration in Zebrafish

**Figure 1:**
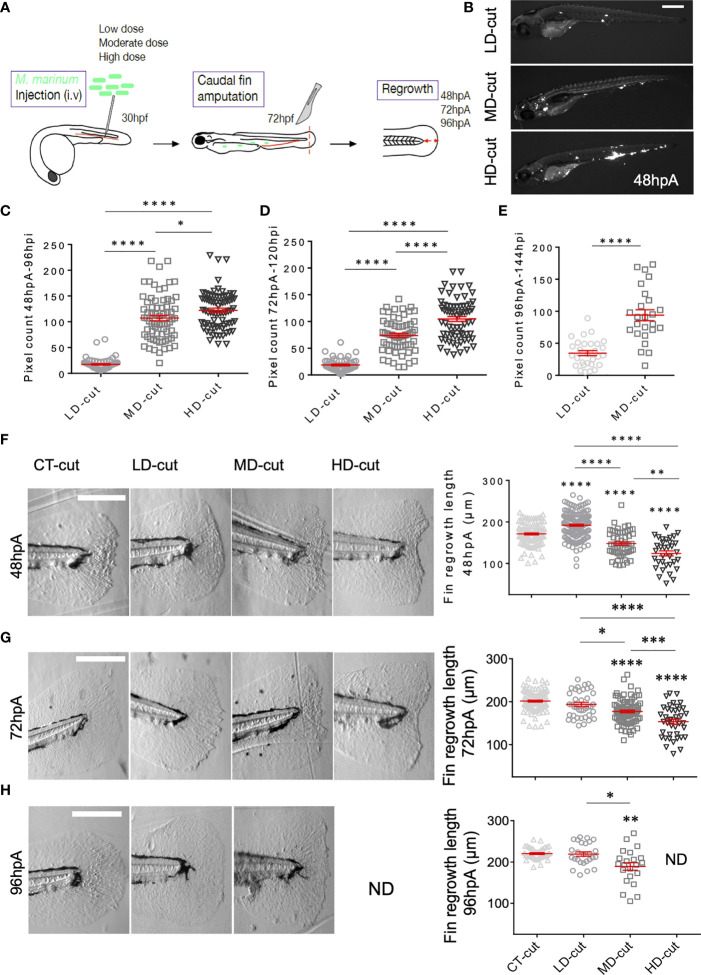
*M. marinum* infection impacts on the regrowth of the caudal fin after amputation. **(A)** Infection and amputation experiment design. **(B)** Whole larvae infected with low (LD), moderate (MD) and high (HD) doses with the Wasabi-expressing *M. marinum* M strain, imaged at 48 hpA (Scale bar = 500 µm). **(C)** Fluorescent pixel counts (FPC) following infection with LD, MD or HD of *M. marinum* at 96 hpi, corresponding to 48 hpA (mean ± SEM, n> 30, ordinary one-way ANOVA, Tukey’s multiple comparisons test, ****p ≤ 0.0001, *p≤ 0.05). **(D)** FPC following infection with LD, MD or HD of *M. marinum* at 120 hpi, corresponding to 72 hpA (mean ± SEM, n> 30, ordinary one-y ANOVA, Tukey’s multiple comparisons test, ****p≤ 0.0001). **(E)** FPC following infection with LD, MD or HD of *M. marinum* at 144 hpi, corresponding to 96 hpA (mean ± SEM, n< 30, Mann Whitney test, two-tailed, ****p≤ 0.0001). All larvae died with a HD infection at this timepoint. **(F)** Representative images of caudal fin regeneration at 48 hpA (Scale bar = 200 µm) with the corresponding graphs showing the fin length after injection of PBS (CT) or infection with LD, MD or HD of *M. marinum* (mean ± SEM, n> 30, ordinary one-way ANOVA, Dunnett’s multiple comparisons test, compared to control except when indicated, **p ≤ 0.01, ****p ≤ 0.0001). **(G)** Representative images of caudal fin regeneration at 72 hpA (Scale bar = 200 µm) with the corresponding graphs showing the fin length after injection of PBS (CT) or infection with infection with LD, MD or HD of *M. marinum* (mean ± SEM, n> 30, ordinary one-way ANOVA, Dunnett’s multiple comparisons test, compared to control except when indicated, *p≤ 0.05, ***p ≤ 0.001, ****p ≤ 0.0001). **(H)** Representative images of caudal fin regeneration at 96 hpA (Scale bar = 200 µm) with the corresponding graphs showing the fin length after injection of PBS (CT) or infection with LD, MD or HD of *M. marinum* (mean ± SEM, n<30, Kruskal-Wallis, Dunn’s multiple comparisons test, compared to control except when indicated, *p≤ 0.05, **p≤ 0.01). All larvae died with the HD infection at this timepoint. ND, not determined.

To evaluate the effect of infection on tissue regeneration, we used the zebrafish caudal fin transection model, a relevant proxy to study the paradigm of appendage regeneration. At 72 hours post-fertilization (hpf), zebrafish regenerate the caudal fin fold in three days (9). Herein, we performed an intravenous (i.v.) injection of three different doses of the fish pathogen *M. marinum* in zebrafish larvae at 30 hpf prior to fin amputation at 72 hpf to study the regeneration process, as outlined in **Figure 1A**. The accuracy of the low, moderate and high doses of *M. marinum* expressing Wasabi was first assessed by microscopic examination of whole infected larvae at 48 hours post amputation (hpA) (**Figure 1B**) and at 72 and 96 hpA (**Supplementary Figures 1A, B**). Fiji (ImageJ software) was subsequently used to quantify the bacterial burden, reflected by the corresponding fluorescent pixel counts (FPC) (15) at 48, 72 and 96 hpA. Our results confirm that the different doses were properly administered into the larvae, as judged by the correlation between the infectious inoculum (low, moderate, high) and the FPC at different time points post-amputation (**Figures 1C–E**). To address whether *M. marinum* infection influences caudal fin fold regeneration, we next evaluated the fin regenerative outgrowth by measuring the length and the area of the regenerating tissue from the initial amputation position to the new distal fin edge. Neither the size nor the morphology of the regenerating fin was affected with the low dose at the early phase of regeneration i.e., at 24 hpA (data not shown). Unexpectedly, at 48 hpA, the low dose was associated with a significant increase in the regenerative fin fold growth (**Figure 1F** and **Supplementary Figure 1D**) that resulted in full regeneration of the caudal fin fold, which is usually not completed until 72 hpA in the uninfected control larvae (**Figure 1F** and **Supplementary Figure 1C**). Contrasting with these findings, injection of moderate or high doses of *M. marinum* significantly retarded fin fold regeneration in a dose-dependent manner at 48 hpA (**Figure 1F** and **Supplementary Figure 1D**), 72 hpA (**Figure 1G** and **Supplementary Figure 1E**) and 96 hpA (**Figure 1H** and **Supplementary Figure 1F**). Measuring the wound contour straightness of the regenerating fin fold in control and infected larvae at 72 hpA revealed that high bacterial doses significantly increased the straightness coefficient, suggesting that the caudal fin did not recover their original rounded shape, thereby confirming the deleterious effect of a severe infection on the regeneration process (**Supplementary Figure 1G**).

To inquire whether the effect observed upon infection on fin fold regeneration depends on the virulence status of *M. marinum*, similar experiments were conducted using a mutant deficient for the RD1 locus, coding for the type VII secretion system ESX-1, conserved in many pathogenic mycobacterial species, such as *M. tuberculosis* (16, 17). Injection of low, moderate and high doses of ΔRD1 led to disseminated infections (**Supplementary Figures 2A, B, E**) with a higher bacterial burden after injection with the moderate and high doses (**Supplementary Figure 2F**). However, ΔRD1 was attenuated as exemplified by the lower bacterial loads as compared to those of the wild-type progenitor (**Figures 1C–E**), as reported earlier (16). In contrast to larvae infected with the wild-type strain, infection with a low dose of ΔRD1 did not accelerate fin regeneration at 48 hpA in terms of regrowth length (**Supplementary Figure 2C**) and area (**Supplementary Figure 2D**). ΔRD1 infection at moderate and high doses also did not impact on the length, area and straightness of the regenerated fin fold at 72 hpA (**Supplementary Figures 2G-I**).

Together, these findings support the hypothesis that low dose infection does not compete with the regeneration process but rather catalyzes/accelerates this paradigmatic process while moderate/severe infection impairs regeneration and that these effects are dictated by the virulence status of the invading pathogen.

### The Dose-Dependent Effect of *M. marinum* on Fin Regeneration Relies on Blastema and Morphogenesis Modifications

**Figure 2:**
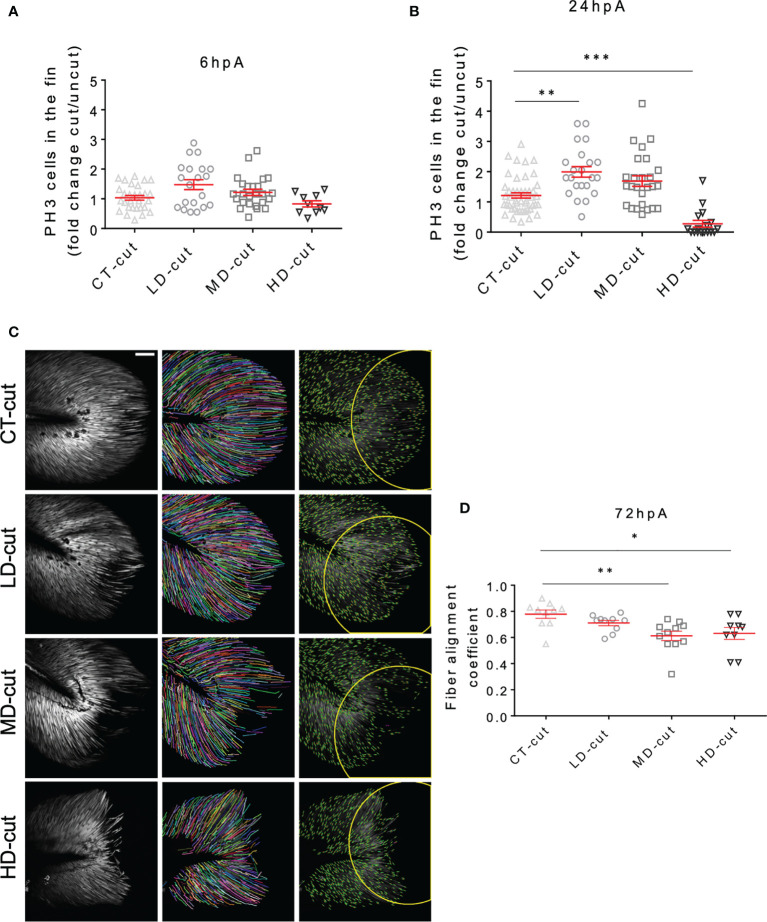
Infection influences cell proliferation, and structure of collagen fibers in the regenerated caudal fin. **(A)** Blastema cellular proliferation after injection of PBS (CT) or LD, MD and HD infection with *M. marinum* prior to caudal fin amputation Anti-PH3 antibody staining of the cells in the fin at 6 hpA, expressed as fold change cut/uncut (mean ± SEM, n< 30, Kruskal-Wallis, Dunn’s multiple comparisons test, non-significant). **(B)** Blastema cell proliferation after injection of PBS (CT) or LD, MD and HD infection with *M. marinum* prior to caudal fin amputation. Anti-PH3 antibody staining of the cells in the fin at 24 hpA, expressed as fold change cut/uncut (mean ± SEM, n< 30, Kruskal-Wallis, Dunn’s multiple comparisons test, **p≤ 0.01, ***p≤ 0.001). **(C)** Second harmonic imaging Z projections were done after injection of PBS (CT) or LD, MD and HD of *M. marinum* at 72 hpA (Scale bar = 60 µm). The procedure of fiber alignment analysis is presented by representative images of the different conditions. The recorded second harmonic images (left) have been subjected to a fiber extraction algorithm (middle) and then the alignment of fiber endpoints (orientation of endpoints are marked with green lines) that were fit inside the fin-width-wide circular region of interests (ROI) (right) were analyzed. **(D)** Fiber alignment in the circular ROI after injection of PBS (CT) or infection with LD, MD or HD of *M. marinum* at 72 hpA (mean ± SEM, n<30, Kruskal-Wallis, Dunn’s multiple comparisons test, *p ≤ 0.05, **p ≤ 0.01).

Caudal fin fold regeneration requires the formation of a heterogenous cellular structure, designated blastema, characterized by a boost of cell proliferation as early as 6 hpA in the region next to the larval stump, followed by propagation of cell proliferation to more proximal regions from 24 hpA (18). We evaluated the proliferative potential of blastemal cells during the regeneration process in *M. marinum*-infected larvae by immunodetection of Phosphorylated Histone 3 (PH3), which labels proliferative cells (10). At 6 hpA, no significant changes in PH3 labelling were observed between the infected *versus* non-infected larvae, regardless of the infectious dose used (**Figure 2A**). However, at 24 hpA, while cell proliferation was significantly increased in zebrafish infected with a low bacterial dose, cell proliferation at the wound was strongly reduced in the high dose-infected zebrafish as compared to the non-infected controls (**Figure 2B**). This indicates that low infection stimulates blastemal cell proliferation at 24 hpA to enhance the regeneration process, consistent with an acceleration of the regenerative growth.

During the embryonic stage, the larva fin fold is mainly composed of mesenchymal cells that modify their shape at the wound site during the regeneration, initially adopting a round shape from 48 hpA and then undergoing elongation and full recovery of their initial shape by 72 hpA (19). Live imaging using *Tg(rcn3:gal4/UAS:DsRed)*, a mesenchymal zebrafish transgenic line (20), allows the identification of the mesenchymal cell pattern and behaviour in the regenerating fin fold. Faster than in the control condition, i.e., at 48 hpA, mesenchymal cells recovered an elongated shape in larvae infected with a low bacterial dose (**Supplementary Figure 3A**). In contrast, at 72 hpA, while mesenchymal cells are elongated and well-organized/aligned in the regenerated control fin, they appear round and disorganized in the fin fold of zebrafish infected with moderate and high bacterial doses as judged by the presence of large areas devoid of mesenchymal cells (**Supplementary Figure 3B**). This suggests that a low bacterial dose promotes the recovery of the original mesenchymal cell shape and organization within the fin while moderate/higher doses impair these criteria. To gain further insights into the impact of *M. marinum* infection on the morphogenesis and fiber content/organization of the regenerated fin fold, second harmonic imaging was performed at 72 hpA (**Figure 2C**). The alignment of fiber endpoints was obtained inside the fin-width-wide circular region of interests. While no noticeable changes in the fiber density and organization of the regenerated fin were observed for the low infectious dose as compared to the control regenerated fin, zebrafish infected with moderate and high doses exhibited pronounced morphological defects (**Figure 2C** and **Movies 1**–**4**). Subsequent fiber alignment analysis showed that, as compared to the uninfected controls, zebrafish infected with a low dose exhibited only a slightly disorganized fiber alignment at 72 hpA. Moderate and high doses led to a severe impairment in the fiber growth and alignment in the amputated caudal fin fold at 72 hpA (**Figures 2C, D**). Measurement of the fiber alignment coefficient at 72 hpA indicated that while the low dose was not associated with a significant deleterious effect, moderate and high doses considerably reduced the alignment coefficient (**Figure 2D**).

Overall, infection of zebrafish with a low dose of *M. marinum* enhances caudal fin regeneration by increasing cell proliferation in the blastema at 24 hpA and accelerates the recovery of mesenchymal cell elongated morphology. Conversely, infection with higher doses impede blastemal cells proliferation at 24 hp and mesenchymal cell organization, ultimately resulting in a disrupted final pattern of mesenchymal cell distribution.

### Low Dose of *M. marinum* Accelerates Macrophage Recruitment in the Early Phase of Regeneration

**Figure 3:**
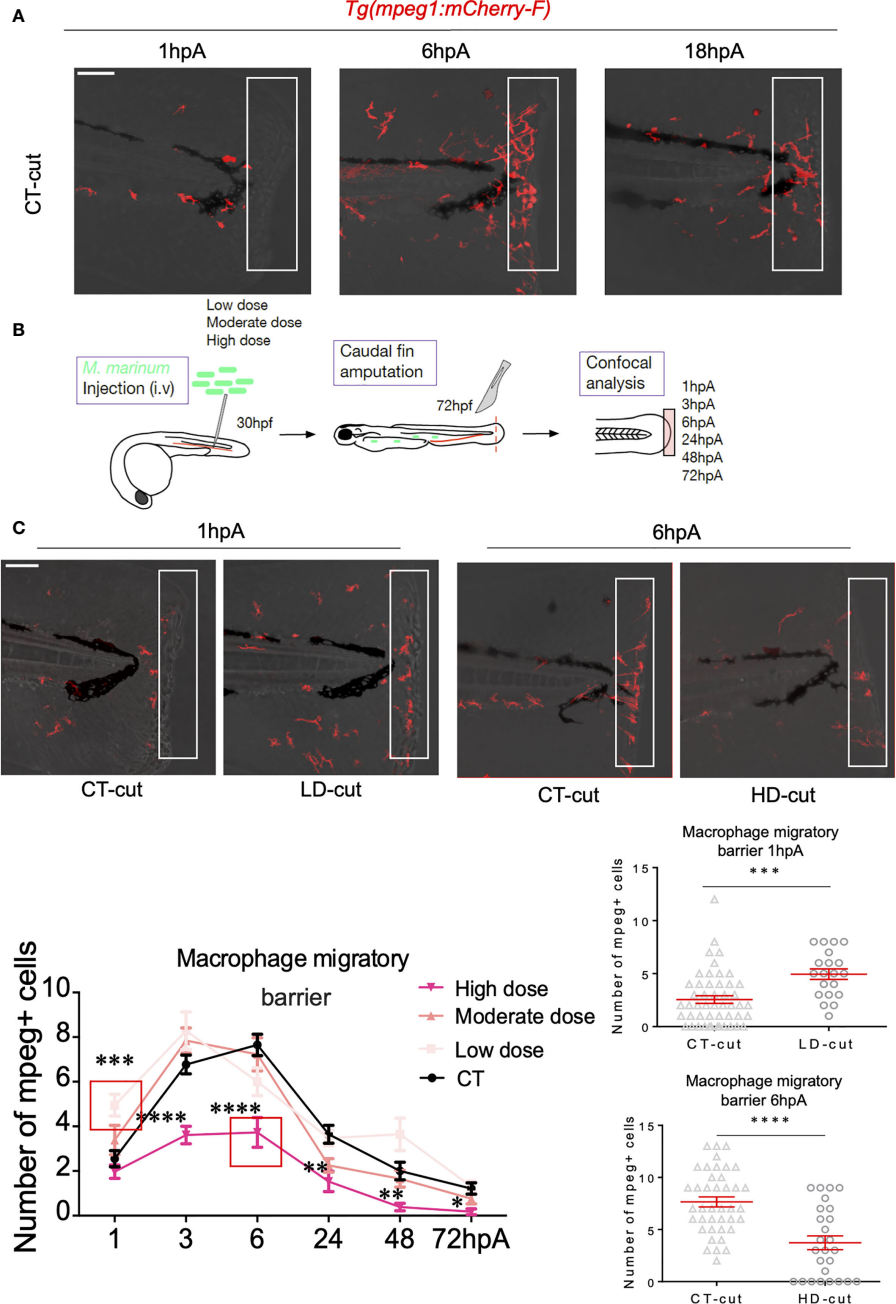
Establishment of the macrophage barrier in the regenerated caudal fin is influenced by the infection. **(A)** Macrophage barrier under non-infected conditions. Confocal images taken at different timepoints after amputation in non-infected *Tg(mpeg1:mCherry-F)* larvae, illustrating arrival of macrophages (1 hpA), positioning of macrophages (6 hpA) and departure of macrophages (18 hpA). **(B)** Experiment design performed in the *Tg(mpeg1:mCherry-F)* line to study the macrophage barrier under infected conditions. **(C)** Kinetic of arrival and departure of *mpeg* ^+^-positive cells, at the caudal fin tip after injection of PBS (CT) or infection with LD, MD or HD of *M. marinum* at 1, 3, 6, 24, 48 and 72 hpA (mean ± SEM, n< 30, Kruskal-Wallis, Dunn’s multiple comparisons test, *p≤ 0.05, **p≤ 0.01, ***p≤ 0.001, ****p≤ 0.0001). Zoom at 1 hpA with Z projections of confocal images illustrating macrophage mobilization in non-infected (CT-cut) larvae or following LD infection at 1 hpA (Scale bar = 60 µm). Quantification of the number of *mpeg* ^+^ cells in the fin tip at 1 hpA after injection of PBS (CT) or infection with LD of *M. marinum* (mean ± SEM, n<30, Kruskal-Wallis, Dunn’s multiple comparisons test, ***p≤ 0.001). Zoom at 6 hpA with Z projections of confocal images illustrating macrophage mobilization in non-infected (CT-cut) larvae or following HD infection at 6 hpA (Scale bar = 60 µm). Quantification of the number of *mpeg* ^+^ cells in the fin tip at 6 hpA after injection of PBS (CT) or infection with HD of *M. marinum* (mean ± SEM, n< 30, Kruskal-Wallis, Dunn’s multiple comparisons test, ****p≤ 0.0001).

Previous work highlighted the importance of a specific macrophage subset response during appendage regeneration (10, 21, 22). Other studies showed that *M. marinum* infection modulates the macrophage response (23). We thus reasoned that the macrophage response may be further modified during the caudal fin fold regeneration in infected larvae. This was interrogated by studying the formation of the macrophage barrier using the zebrafish *Tg(mpeg1:mCherry-F)* transgenic line, harbouring red fluorescent macrophages (24). Embryos at 30 hpf were infected with low, moderate and high doses of *M. marinum* prior to caudal fin transection at 72 hpf and macrophages were analysed by fluorescence microscopy between 1 and 72 hpA. In the absence of infection, at 6hpA, macrophages migrated to the amputated fin and position themselves in a well-organized line at the site of amputation (**Figure 3A** and **Movies 5**–**7**). In zebrafish infected with a low bacterial dose (**Figure 3B**), macrophages aligned themselves at the amputation site prematurely i.e., at 1 hpA, which was not observed with the high infectious dose, even at 6 hpA (**Figure 3C**). This prompted us to checked the distribution of macrophages in whole larvae without amputation at 1hpA, since a large number of macrophages could leave the caudal hematopoietic tissue (CHT) during the infection, possibly explaining the earlier recruitment of macrophages forming a barrier after amputation in the low dose infection. **Supplementary Figure 3C** failed to show major differences between the low dose and the uncut condition at 1hpA. However, the distribution of macrophages in moderately and highly infected conditions appeared more heterogenous, presumably because these infectious doses lead to the formation of granuloma-like aggregated macrophages throughout the whole larvae (**Supplementary Figure 3C**).

When repeating these experiments with zebrafish infected with a low dose of ΔRD1, no changes in the kinetic of the formation of the macrophage barrier were seen at 1 hpA, suggesting that the low dose effect is directly linked to the virulence of the *M. marinum* wild-type strain (**Supplementary Figure 4A**). Similarly, moderate or high doses of ΔRD1 failed to alter the early recruitment of macrophages to the barrier (**Supplementary Figure 4A**). Conversely, a massive disruption of the macrophage barrier was seen in the amputated fin of zebrafish infected with the high dose of the wild-type strain. This correlated with an important decrease in the total number of macrophages recruited at the edge of the wound of highly infected fish between 3 and 72 hpA (**Figure 3C**). In agreement with previous findings in the entire fin (8, 22), we found that in uninfected animals, macrophages (*mpeg1* ^+^) were rapidly recruited to the wound and remained present in the regenerating fin from 1 hpA until complete regeneration at 72 hpA, while peaking at 6 hpA (**Figure 3C**). Whereas the number of macrophages recruited at the wound edge site at 1 hpA was significantly higher with the low dose infection, this was not the case with the high infectious dose, characterized by a pronounced decrease in macrophage recruitment during the entire regeneration process (**Figure 3C**).

Together, these results highlight the opposite effect of low *versus* moderate/high dose infection on caudal fin regeneration, correlating with a differential macrophage migratory behaviour and recruitment at the amputation site.

### *M. marinum* Infection Skews the Macrophage Response During Regeneration

**Figure 4:**
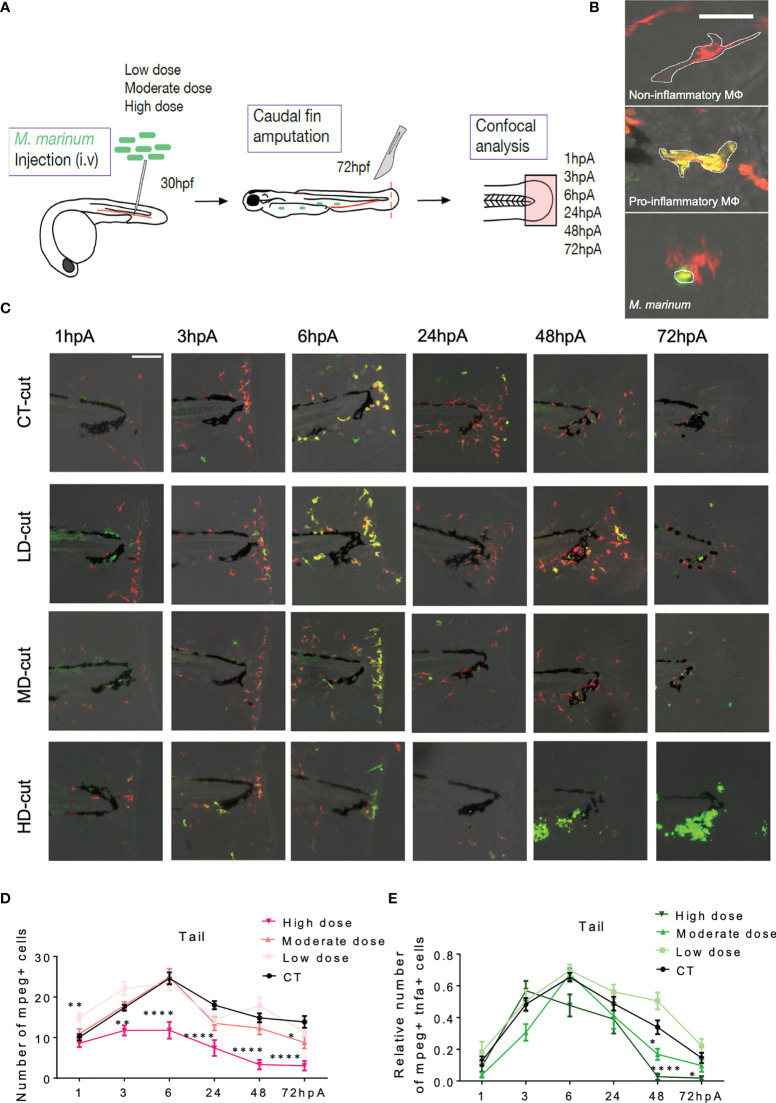
**(A)** Experimental design of macrophage recruitment, and activation following infection and amputation. **(B)** Legends (Z projections of confocal images) to distinguish non-inflammatory macrophages (red) in *Tg(mpeg1:mCherry-F)* larvae from pro-inflammatory (orange) macrophages in (*Tg(mpeg1:mCherry-F;tnfa:eGFP-F)* larvae (Scale bar = 30 µm). Wasabi-expressing *M. marinum* are in green. **(C)** Z projections of confocal images allowed to establish the kinetic of recruitment and activation of macrophages after injection of PBS (CT) or infection with LD, MD or HD of *M. marinum* at 1, 3, 6, 24, 48, 72 hpA (Scale bar = 100 µm). **(D)** Kinetic of total macrophages recruited after fin amputation following injection of PBS (CT) or infection with LD, MD or HD of *M. marinum* (mean ± SEM, n*<* 30, Kruskal-Wallis, Dunn’s multiple comparisons test, *p ≤ 0.05, **p ≤ 0.01, ****p ≤ 0.0001). **(E)** Kinetic of pro-inflammatory macrophages recruitment (expressed as the fold change of *mpeg^+^* and *tnfa^+^* macrophages over the total number of *mpeg^+^* macrophages) after fin amputation following injection of PBS (CT) or infection with LD, MD or HD of *M. marinum* (mean ± SEM, n*<*30, Kruskal-Wallis, Dunn’s multiple comparisons test, *p ≤ 0.05, ****p ≤ 0.0001).

Sequential kinetic of recruitment and activation of different macrophage subsets occurs during the regeneration process in the entire fin (10). While pro-inflammatory macrophages positive for *tnfa*, a marker of M1 macrophages, accumulate during the early phase of regeneration, anti-inflammatory macrophages peak at the later stages during fin regeneration (10). To investigate whether the different infection conditions alter the kinetic of macrophage subsets recruitment and activation, we took advantage of the *Tg(mpeg1:mCherry-F/tnfa:eGFP-F)* zebrafish line, followed by confocal microscopy analysis at 1, 3, 6, 24, 48 and 72 hpA (**Figure 4A**). In this transgenic line, all macrophages express a farnesylated mCherry (mCherry-F) while pro-inflammatory macrophages express *tnfa* along with mCherry-F and farnesylated eGFP (GFP-F) (**Figure 4B**) (9). Focusing exclusively on macrophages in the entire regenerating caudal fin fold (and not only at the wound edge), we consistently observed that, in low dose infected zebrafish, the total number of macrophages in the regenerating fin was significantly higher at 1 hpA as compared to the non-infected controls (**Figures 4C, D**). In comparison, fish infected with a low dose of ΔRD1 did not show any significant differences as compared to the non-infected controls (**Supplementary Figure 4B**), further supporting that the low dose effect is connected with *M. marinum* virulence. In contrast, in the high dose infected fish, the total number of macrophages in the fin was significantly lower from 3 hpA and maintained during the entire regeneration period (**Figures 4C, D**). This was associated with a reduced accumulation of pro-inflammatory macrophages expressing *tnfa* in the regenerating fin of zebrafish inoculated with a moderate dose at 48 hpA and a high dose at 48 and 72 hpA, as compared to uninfected controls (**Figures 4C–E**).

We hypothesized that the impaired macrophage recruitment to the amputation site of zebrafish inoculated with a high dose was associated with a reduced overall macrophage pool. The total number of macrophages in *Tg(mpeg1:mCherry-F)* zebrafish was quantified by flow cytometry after dissociation of the whole larvae. No significant differences were found between the different infected conditions at 48 hpA (**Supplementary Figure 5A**). Despite these equivalent numbers of macrophages, these seemed to be preferentially present at sites other than the wound site in the heavily infected conditions. Supporting this view, we found that the high dose was associated with increased granuloma formation, consisting mainly of aggregated macrophages in the whole larvae at 48 hpA (**Supplementary Figure 4C**). This suggests that macrophages are preferentially attracted or retained to areas outside the amputation site, particularly in granulomas. However, at 72hpA, a near-complete disappearance of macrophages occurred in the whole larvae as revealed by epifluorescence microscopy (**Supplementary Figure 4D**) and confocal microscopy (data not shown), regardless whether the fin was amputated or not.

Next, we asked whether the disappearance of macrophages in highly infected zebrafish, at 72hpA, was associated with the capacity of *M. marinum* to regulate the expression of *mpeg1*, as suggested previously (25). This was achieved by injecting high doses of *M. marinum* in two zebrafish lines, *Tg(mpeg1:mCherry-F)* and *Tg(mfap4:mCherry-F)*, allowing to track all macrophages using two distinct markers, *mpeg1* and *mfap4*, respectively. Epifluorescence microscopy revealed a comparable disappearance of the macrophages in both transgenic reporter lines at 72 hpA in whole larvae (**Supplementary Figure 4E**) and in the caudal fin (**Supplementary Figure 4F**). Subsequent confocal microscopy imaging and quantification did not show significant differences in the total number of macrophages at 48 and 72 hpA in the fin of both transgenic lines (**Supplementary Figures 4G, H**). This excludes the possibility that the disappearance of macrophages at the late stage of regeneration may result from a decreased expression of *mpeg1*.

Collectively, these results demonstrate that high dose infection with *M. marinum* not only disrupts macrophage accumulation at the wound site of regenerating fish, but also impairs their activation and polarization towards a pro-inflammatory phenotype. These findings also suggest that the tightly regulated macrophage response, pivotal for the regeneration process (10), is dysregulated in the context of a severe infection, and results in macrophage depletion at 72 hpA in the whole zebrafish and at the wound site.

### Infection With *M. marinum* Alters Macrophage Viability in the Regenerated Caudal Fin

**Figure 5:**
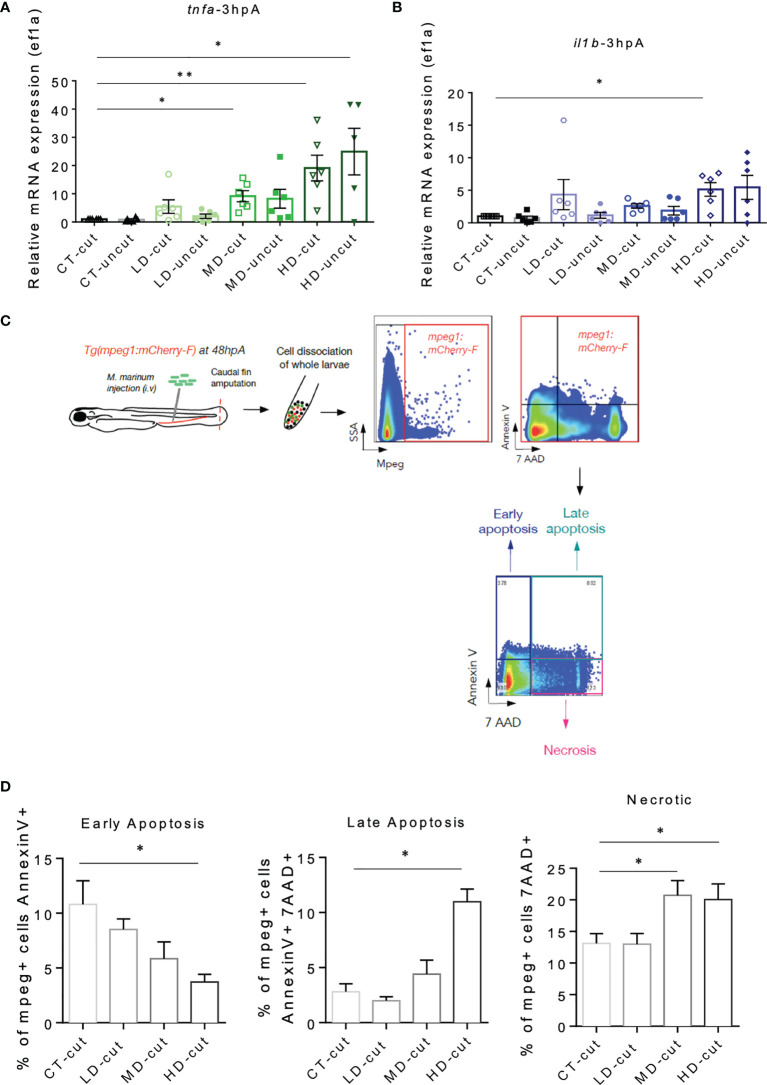
*M. marinum* infection impacts on macrophages viability in the regenerated caudal fin. **(A)** Expression of *tnfa* transcripts in uncut and cut caudal fins after injection of PBS (CT) or infection with LD, MD or HD of *M. marinum* at 3 hpA. Analysis was performed by RT-qPCR and results are expressed as the *tnfa/ef1a* ratio (mean ± SEM, n=5-6, Kruskal-Wallis, Dunn’s multiple comparisons test, *p ≤ 0.05, **p ≤ 0.01). **(B)** Expression of *il1b* transcripts in uncut and cut caudal fins after injection of PBS (CT) or infection with LD, MD or HD of *M. marinum* at 3 hpA. Analysis was performed by RT-qPCR and results are expressed as the *il1b/ef1a* ratio (mean ± SEM, n = 5-6, Kruskal-Wallis, Dunn’s multiple comparisons test, *p≤ 0.05). **(C)** Experimental design to study macrophage viability using flow cytometry. **(D)** Percentage of total apoptotic and necrotic macrophages (mpeg^+^ cells) in the whole larva after injection of PBS (CT) or infection with LD, MD or HD of *M. marinum* at 48 hpA was obtained by flow cytometry (mean ± SEM, n = 4-5, Mann Whitney test, one tailed, *p ≤ 0.05).

TNF has been shown to cause necrosis of macrophages infected with *M. marinum* in zebrafish (26), which may explain the decreased number of macrophages observed at the wound site and in the whole zebrafish larva with a high infectious dose at 72 hpA. Quantification of pro-inflammatory macrophages expressing *tnfa* failed to show an increased frequency of this macrophage subset in the early phase of regeneration (**Figure 4E**). Thus, we wondered whether the pro-inflammatory macrophages accumulating at the wound of zebrafish infected with *M. marinum* would express higher amount of *tnfa* prior to their disappearance, presumably resulting in necrosis. RT-qPCR analysis at 3 hpA identified a higher expression level of *tnfa* in the whole zebrafish infected with moderate and high doses of *M. marinum* as compared to either the untreated or the low dose-treated larvae (**Figure 5A**). There were equal *tnfa* expression levels found in the cut *versus* uncut categories, suggesting that *tnfa* expression relies primarily on infection rather than on amputation at 3 hpA. The expression profile of *il1b*, another pro-inflammatory cytokine that triggers a distinct cell death pathway, termed pyroptosis (27) was also determined. Our results highlight a significant increase in *il1b* expression in the high dose-treated larvae at 3 hpA (**Figure 5B**). To address whether the increased expression of *tnfa* and *il1b* was responsible for the reduced macrophage survival, flow cytometry was performed on *mCherry-F* ^+^ cells after cell dissociation from the entire *Tg(mpeg1:mCherry-F)* larvae at 48 hpA, using combined staining with annexin V and 7-AAD (28) (**Figure 5C**). Annexin V staining labels cells exhibiting early and late apoptosis since it binds with phosphatidylserine translocated from the inner leaflet to the outer leaflet of the plasma membrane of apoptotic cells. 7AAD intercalates into the DNA of cells that have lost their membrane integrity, such as cells in late apoptosis (annexin V- and 7AAD-positive) and necrotic cells (7AAD-positive) but does not penetrate the cells in early apoptosis (annexin V-positive) (28). Thus, while early and late apoptosis were not significantly affected in the macrophages of regenerating larvae pre-infected with a moderate dose of *M. marinum*, the percentage of necrotic macrophages was significantly increased compared to the control (**Figure 5D** and **Supplementary Figure 5B**). Moreover, while the proportion of early apoptotic macrophages in the high dose *M. marinum*-infected zebrafish was reduced, the percentage of late apoptotic and necrotic macrophages was significantly increased as compared to the controls (**Figure 5D**).

Altogether, these results suggest that the exacerbation of the *tnfa* and *il1b* pro-inflammatory response at the beginning of the regeneration process leads to macrophages necrosis in the entire larvae at 72 hpA.

## Discussion

The present study demonstrates that mild infection with *M. marinum* catalyzes/accelerates the regeneration process while severe infection inhibits regeneration and that this process is dependent on the virulence status of the pathogen. Infection of zebrafish larvae with a low dose increases blastema proliferation at 24 hpA during caudal fin regeneration, leading to the formation of a regenerated tissue displaying morphogenesis features similar to those in the non-infected larvae. Conversely, high dose infection significantly alters proliferation of blastemal cells at 24 hpA as well as morphogenesis of the newly formed tissue. The impact of *M. marinum* infection on the regeneration potential is, at least partly, due to differential macrophage responses. Since pro-inflammatory macrophages activate blastema cell proliferation and prime regeneration in zebrafish through the release of TNF (10) and because *M. marinum* infection modulates the macrophage response and TNF production (23), we investigated how macrophages respond and influence the regeneration process, following mycobacterial infection.

**Figure 6:**
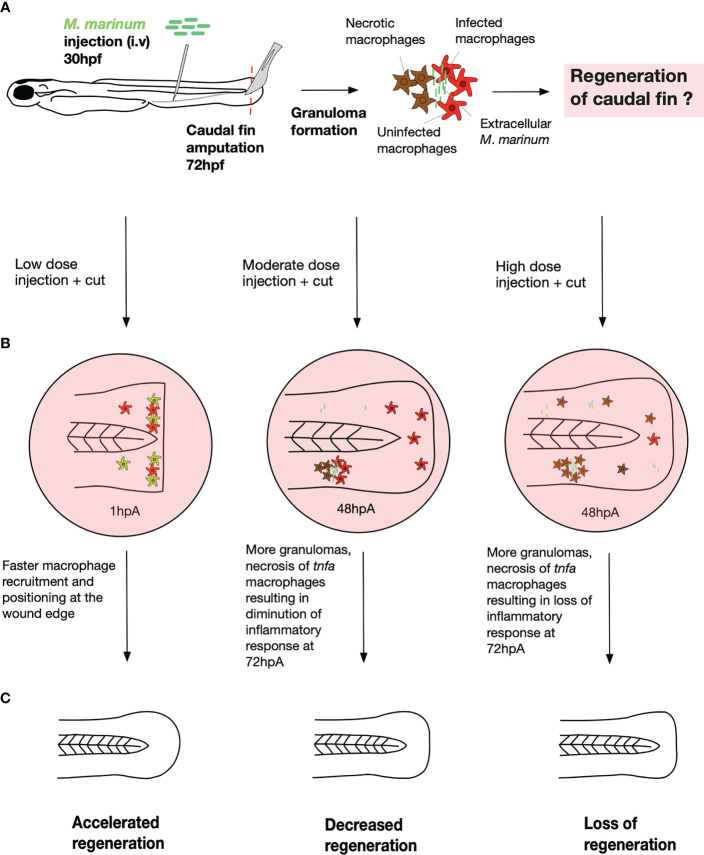
A model describing the macrophage response during the caudal fin regeneration of zebrafish larvae infected with *M. marinum*. **(A)** Infection and amputation experiment design. Injection of low, moderate and high doses of *M. marinum* in the caudal vein at 30 hpf then amputation of the caudal fin at 72 hpf. **(B)** Schematic representation of the mycobacteria dose effect on the macrophage response. The low dose accelerated the macrophage response from 1 hpA as revealed by the more rapid macrophage recruitment and alignment at the fin tip. The moderate dose triggered more granuloma formation and necrosis of macrophages leading to a decreased inflammatory response at 72 hpA. The high dose also caused more granuloma formation, necrosis of macrophages resulting in the absence of macrophage response at 72 hpA. **(C)** Schematic representation of the mycobacteria dose effects on the caudal fin regrowth. The regeneration process was accelerated with the low dose, decreased with the moderate dose and completely lost with the high dose.

In the regenerating fin of the low dose infected larvae, the total number of macrophages increased rapidly after amputation as compared to the non-infected zebrafish (**Figure 6**). Contrasting with these findings, in the high dose infected fish, the total number of macrophages in the fin was drastically reduced during the last 24 hours of the regenerative process as compared to the non-infected zebrafish. The decreased number of macrophages within the fin paralleled an increased number of granulomas at 48 hpA in the entire larva, suggesting that macrophages were preferentially channeled at the site of infection rather than at the site of injury (**Figure 6**). However, an almost complete macrophage depletion occurred at 72 hpA in the whole larvae and the fin fold.

This phenotype was associated with a reduced proportion of pro-inflammatory macrophages expressing *tnfa* between 48 and 72 hpA in the regenerating fin of zebrafish inoculated with a high dose of *M. marinum*. We previously showed that the regeneration process relies on a tightly regulated inflammatory response, typified by i) a transient accumulation of pro-inflammatory macrophages providing TNF-α signaling at the wound site during the early phase of regeneration and creating a permissive environment for the formation of the blastema and ii) an accumulation of anti-inflammatory macrophages during the late phase or regeneration, critical for the fin structure (10). Severe infection with *M. marinum* is characterized by a marked decreased in the number of total and pro-inflammatory macrophages in the fin and the whole larva and inhibits the regeneration process while mild infection sustains a transient macrophage recruitment and accelerates regeneration. However, these results contrast with a previous study describing how infection with *Listeria monocytogenes* affects healing by applying the bacteria in the caudal fin transection model (29). This study unraveled a persistent inflammation with an excess of pro-inflammatory macrophages and a loss of vimentin-positive mesenchymal cells, responsible for an impaired tissue repair process (29). The discrepancies between this study and ours might be due to the fact that i) *L. monocytogenes* infection was performed during fin transectioning, ii) the pathogen doses used were different and iii) *L. monocytogenes* and *M. marinum* likely have different virulence networks and different interactions with the inflammatory response. Furthermore, these two studies address fundamentally different questions about the role of infection and inflammation in the regenerative process, thus emphasizing the complex interplay between host-pathogen interactions in modulating host regeneration.

We showed that macrophages from larvae infected with a high dose of bacteria disappear almost completely throughout the larva at 72hpA. This raises questions about how macrophages disappear from whole larvae infected with high dose of *M. marinum*. A large body of evidence support the view that, like *M. tuberculosis*, *M*. *marinum* activates the inflammasome, a multimeric complex that detects pathogens in macrophages (30–32). Subsequently, the inflammatory cascade leading to the secretion of IL-1β, triggers a distinct cell death pathway, termed pyroptosis (27, 33). Similarly, excessive TNF production leads to necrosis of mycobacteria-infected macrophages (26). In line with these studies, we provide evidence that the disappearance of macrophages in highly infected zebrafish occurs sequentially, following exacerbated *tnfa* and *il1b* expression, early in the regeneration process, resulting in macrophage necrosis and loss of the regeneration process. TNF-α and IL-1β pro-inflammatory cytokines are crucial during the zebrafish regeneration process but their expression/release need to be tightly controlled to completely restore the damaged tissues. Therefore, uncontrolled expression of inflammatory cytokines following *M. marinum* infection impairs the macrophage response and larval regenerative potential.

Macrophages are known to be involved in efficient and dysregulated granuloma turnover with host-beneficial and deleterious effects, respectively. Indeed, the dynamic development of granulomas might be protective for the host or beneficial for mycobacteria since they utilize macrophages as a niche for their own growth and dissemination after necrosis of infected macrophage and granuloma disruption (34–36). One may hypothesize that the disappearance of macrophages by necrosis at high doses of bacteria at 72 hpA promotes bacterial expansion, notably due to loss of granuloma control present in large numbers at 48 hpA and this uncontrolled infection would explain the premature larval killing mortality at 96 hpA (data not shown). Treatments limiting the increased expression of *tnfa* (and macrophage necrosis) could therefore be interesting both to preserve the protective role of granulomas and control infection, but also to promote regeneration.

In our model of systemic infection, we observed that macrophages have a preferred tropism for granulomas rather than for the injured site at 48hpA but additional studies are needed to study the possible impact of these granulomas on regeneration. Localized *M. marinum* infections, for instance in the muscle or hindbrain ventricle, would be helpful to address this question.

An unaddressed question relies on whether the injury affects the bacterial burden in our model of infection. Interestingly, Schild and al. (37) recently showed increased bacterial loads in an experimental design similar to ours, likely indicating that fin amputation influences the infection outcome.

In summary, we have shown that depending on the size of the infectious dose, infection and regeneration do not necessarily compete for monopolizing the macrophages at the injured site (**Figure 6**). On the one hand, a synergistic effect occurs between fin fold injury and the low dose infection that hastens tissue regeneration. On the other hand, a deleterious effect on fin regeneration was observed with the high dose infection. Zebrafish infection with a high dose of *M. marinum* induces granuloma formation, contributing to diverting macrophages from the site of injury. At later stages, necrosis occurs, leading to a near-complete loss of macrophages at the end of the regeneration process, associated with uncontrolled bacterial replication and disseminated infections. This followed an early exacerbation of *tnfa* and *il1b* expression level during the regeneration process. Taken together, our results emphasize the importance to study the regeneration process under infectious conditions in the zebrafish model, aiming at improving the cellular response in infected human wounds.

## Materials and Methods

### Ethics

Animal experimentation procedures were carried out according to the European Union guidelines for handling of laboratory animals (http://ec.europa.eu/environment/chemicals/lab animals/home en.htm) and were approved by the Ministère de l'Enseignement Supérieur de la Recherche et de l'Innovation and Comité d’Ethique pour l’Expérimentation Animale under reference 2020022815234677 V3.

### Zebrafish Lines, Maintenance and Handling

Fish and larva maintenance, staging and husbandry were performed as previously described (10, 38). Embryos were obtained from the University of Montpellier. Experiments were done using golden strain and the following transgenic lines: *Tg(mpeg1:mCherry-F)^ump2^* referred to as *Tg(mpeg1:mCherry-F), Tg(mfap4:mCherry-F)^ump6^* referred to as *Tg(mfap4:mCherry-F)* to visualize macrophages (39); *Tg(mpeg1:mCherry-F)^ump2^* and *Tg(tnfa:GFP-F)^ump5^* referred to as *Tg(mpeg1:mCherry-F;tnfa:eGFP-F)* to visualize *tnfa* expression in macrophages (9); *Tg(rcn3:gal4)^pd1023^* and *Tg5(UAS:mCherry)^pd1112^* referred to as *Tg(rcn3:gal4/UAS:mCherry)* to label mesenchymal cells (40). Embryos were obtained from adult zebrafish pairs by natural spawning and were raised at 28.5 C in zebrafish tank water.

### Bacterial Strains and Culture Conditions

*Mycobacterium marinum* M strain is a human isolate that has been extensively characterized (41). The ΔRD1 mutant, consisting of the M strain lacking the major virulence RD1 locus has been reported elsewhere (16). Bacterial cultures were grown and maintained on Middlebrook 7H10 agar enriched with 10% oleic acid, albumin, dextrose and catalase (OADC; BD Difco) at 30°C or grown in Middlebrook 7H9 broth supplemented with 10% OADC and 0.025% Tyloxapol (Sigma-Aldrich). Green fluorescent *M. marinum* expressing Wasabi were generated after transformation of *M. marinum* with pTEC15 (42) and selection in the presence of 50 µg/mL hygromycin.

For use in zebrafish infection experiments, *M. marinum* M strain (wild-type) and ΔRD1 harbouring pTEC15 were grown in Middlebrook 7H9 liquid broth for 7 days, after which bacteria were processed to generate single cell suspensions as previously described (15, 24). Aliquots of single cell suspensions were frozen and kept at -80°C until further use.

### Infection of Zebrafish Larvae

Bacterial aliquots were thawed and diluted with PBS and phenol red dye (0.5%, w/v) to OD_600_ 1. Fluorescent green *M. marinum* and its RD1 derivative were microinjected into the caudal vein of embryos anesthetized with 0.016% Tricaine at 30 hours post fertilization (hpf) previously dechorionated (13) and at different infectious doses: 30-50 (low dose), 150-200 (moderate dose), and 500-700 (high dose) colony forming units (CFU). The bacterial inoculum was checked *a posteriori* by plating 2 nL of the bacterial suspension on Middlebrook 7H10 and CFU determination after 7 days of incubation at 30°C. Following infection, larvae were transferred into E3 media at 28.5°C.

### Larvae Manipulation for Regeneration Assays

Caudal fin amputation was performed on 72 hpf larvae, as previously described (9), equivalent to 48 hrs post-infection (hpi). Larvae were anaesthetized in embryo medium supplemented with 0.016% Tricaine and the caudal fin was amputated with a microbial-free scalpel at the limit of the notochord posterior end.

### Imaging of Larvae and Quantification

For imaging, larvae were anesthetized in 0.016% Tricaine, immobilized in 35 mm glass bottom dishes (FluoroDish™, World Precision Instruments) using 1% low melting point agarose (Sigma-Aldrich) and covered with a small volume of fish water containing tricaine. Epifluorescence microscopy was used to quantify bacterial loads and measure the length and the area of regrowth and performed with a Zeiss Axio Zoom.V16 equipped with an Axiocam503 monochrome (Zeiss) camera. Fluorescent pixel count (FCP) determination, which reflects the bacterial loads, was performed with Fiji (ImageJ Software), using the ‘Analyze particles’ function (15). Regenerative fin growth length was evaluated by measuring the distance between the amputation plane and the edge of the fin in the median plane with Fiji. Regenerative fin fold area was measured as the fold area between the notochord and the edge of the fin using the polygon tool on Fiji. The straightness of the contour length of the fin was determined using the segmented line selection function in Fiji. The contour of the fin was drawn three times on each caudal fin to limit manipulator error, then the shortest distance (obtained using Feret’s diameter) and the actual length between the endpoints of the selection boundary was recovered. The fold change between the feret’s and actual lengths were calculated to determine the rectitude of the fins (rectilinear: close to 1). Confocal microscopy was employed to study macrophage barrier, recruitment and activation, using an inverted confocal microscope TCS SP5 and TCS SP8 MP on the Cartigen Plateform from IRMB (Leica Microsystems). Images were taken in a sequential mode by frame. Image stacks for time lapse videos were acquired every 5 min, scanning at 5 µm intervals at a 1024x1024 pixel resolution. The 4D files generated from time lapse acquisitions were processed using Fiji, compressed into maximum intensity projections and cropped. Brightness, contrast, and color levels were adjusted for maximal visibility. The macrophage barrier was observed with *Tg(mpeg1:mCherry-F)* between 15 min post-amputation (mpA) and 72 hpA and recruitment and activation of macrophages were tracked with *Tg(mpeg1:mCherry-F;tnfa:eGFP-F)* taking images at 1, 3, 6, 24, 48, and 72 hpA. Recruited and activated macrophages were counted directly on microscopy images using Fiji.

### Second Harmonic Imaging Microscopy and Collagen Fiber Analysis

Second harmonic generation (SGH) imaging was performed on fixed caudal fin samples from golden strain (43–45). The caudal fin was amputated from the larvae with a scalpel, and the larvae was then placed on a glass cavity slide in a drop of PBS covered with a coverslip to minimize movement. Caudal fins were imaged using a custom-made multiphoton microscope built on a Tsunami tunable Ti : Sapphire laser (Spectra-Physics) and an upright SliceScope microscope (MPSS-1000P, Scientifica) equipped with a galvanometer scan head (MP-2000, Scientifica) using a Nikon 16x long working distance water immersion objective (CFI75 LWD-16x-W). The sample was excited with a 760-900 nm wavelength range laser light operating the Ti : Sapphire laser in pulsed mode configuration at 80 MHz frequency and ~100 fs pulse duration. SHG signal detection was done in transmission by a H7422P photomultiplier (Hamamatsu) using a 1.4-NA oil-immersion condenser (U-AAC, Olympus), a 482 nm long pass dichroic mirror (86-331, Edmund Optics) and a 447 nm high-performance band-pass filter (48-074, Edmund Optics) setup. Z-stack images were recorder in 1024x1024 and 512x512 pixels resolutions with 1 µm spacing between image planes at 200 line/s scanning rate.

Preprocessing of the recorded raw z-stack images was done in Fiji (46), including slice averaging, noise reduction and z-projections for further fiber analysis and 3D-projections for visualization (**Movies 1**–**4**).

Collagen alignment quantification was performed on z-projections of the recorded z-stacks by CurveAlign 4.0, an open source fibrillar collagen quantification platform (https://eliceirilab.org/software/curvealign/) (47, 48).

### Cell Proliferation

Proliferative cells were labelled using immunodetection with anti-phosphorylated histone 3 antibody (PH3). At 6 and 24 hpA, larvae were fixed in 4% paraformaldehyde overnight at 4°C and stained as previously described (9) using the Rabbit anti-P3H antibody (Cell Signaling). Positive cells in the fin region were quantified on confocal images using Fiji.

### RNA Preparation on Larvae and Quantitative RT-PCR

To determine the relative expression of *tnfa* and *il1b* normalized with housekeeping gene *ef1a*, total RNA from whole larvae (pools of 10 individuals per group) were prepared at 3 hpA. RNA preparation and reverse transcription were performed, as described previously (10). RT-qPCR analyses were performed using the Light Cycler 480 system software.

### Flow Cytometry on Zebrafish Cells

Groups of 40 larvae (*Tg(mpeg1:mCherry-F)* were infected with *M. marinum* and amputated for each conditions. Cells from larvae were dissociated at 48 hpA, as previously described (9). To monitor membrane permeability and identify cells undergoing necrosis, dye 7-amino-actinomycin D (7AAD) (BD Pharmigen) was used. To monitor early apoptosis, the Annexin V FITC dye was used (BD Pharmigen 556547). Apoptosis and necrosis of macrophages were quantified using flow fluorocytometry (Symphony, Cartigen plateform IRMB).

### Statistical Analyses

Graph Pad Prism 6.0 Software (San Diego, CA, USA) was used to generate the graphs and to analyze the data. Specific statistical tests were used to evaluate the significance of differences between the groups. Graphs show the mean ± standard error of the mean (SEM). Student’s *t-*tests were performed to compare the significance levels of two groups with n>30. The Mann-Whitney test was performed to compare the significance levels of two groups with n<30. The ANOVA test was performed to compare more than two groups with n>30. The Kruskal-Wallis ANOVA was performed to compare more than two groups with n<30, except for the **Figure 5C** for which a Mann-Whitney one-tail test was applied.

## Data Availability Statement

The original contributions presented in the study are included in the article/**Supplementary Material**. Further inquiries can be directed to the corresponding author.

## Ethics Statement

The animal study was reviewed and approved by APAFIS#24406-2020022815234677v3

## Author Contributions

CB and FD designed experiments with input from MJ and LK. CB, MJ, BV, RC-L, AB, and CH performed experiments. FD and CB wrote the manuscript with input from DS, CJ, MJ, and LK. All authors contributed to the article and approved the submitted version.

## Funding

This work was supported by INSERM and the University of Montpellier. We also thank Foundation for Research in Rheumatology (FOREUM; SEN-OA) and “Agence Nationale de Recherche” (ANR-18-CE18-0010) for financial support of this project. MJ received a post-doctoral fellowship granted by Labex EpiGenMed, an «Investissements d’avenir» program (ANR-10-LABX-12-01).

## Conflict of Interest

The authors declare that the research was conducted in the absence of any commercial or financial relationships that could be construed as a potential conflict of interest.

## Publisher’s Note

All claims expressed in this article are solely those of the authors and do not necessarily represent those of their affiliated organizations, or those of the publisher, the editors and the reviewers. Any product that may be evaluated in this article, or claim that may be made by its manufacturer, is not guaranteed or endorsed by the publisher.

## Supplementary Material

Supplementary Figure 1 Caudal fin regrowth is conditioned by the severity of the infection. **(A)** Representative images of whole larvae infected with LD, MD or HD of *M. marinum* expressing Wasabi, at 72 hpA (Scale bar = 500 µm). **(B)** Representative images of whole larvae infected with LD, MD or HD of *M. marinum* expressing Wasabi, at 96 hpA (Scale bar = 500 µm). All larvae died with the HD inoculum at 96 hpA. **(C)** Graph represents the kinetic of fin length after injection of PBS (CT) or LD of *M. marinum*, between 48 and 72 hpA (mean ± SEM, n>30, t-test, two-tailed, **** p<0.0001). **(D)** Graphs showing the fin area after injection of PBS (CT) or infection with LD, MD or HD of *M. marinum* at 48 hpA (mean ± SEM, n> 30, ordinary one-way ANOVA, Dunnett’s multiple comparisons test, compared to control except when indicated, ****p ≤ 0.0001). **(E)** Graphs showing the fin area after injection of PBS (CT) or infection with LD, MD or HD of *M. marinum* at 72 hpA (mean ± SEM, n>30, ordinary one-way ANOVA, Dunnett’s multiple comparisons test, compared to control except when indicated, ****p ≤ 0.0001). **(F)** Graphs showing the fin area after injection of PBS (CT) or infection with LD, MD or HD of *M. marinum* at 96 hpA (mean ± SEM, n<30, Kruskal-Wallis, Dunn’s multiple comparisons test, compared to control except when indicated, ****p ≤ 0.0001). All larvae died with the HD infection at this timepoint. ND, not determined. **(G)** Graphs represent the wound contour straightness of the fin after injection of PBS (CT) or LD, MD and HD of *M. marinum*, at 72 hpA (mean ± SEM, n>30, ordinary one-way ANOVA, Dunnett’s multiple comparisons test, ****p≤ 0.0001). Explanatory diagram.

Supplementary Figure 2 Deletion of RD1 abrogates the effect on fin regeneration. **(A)** Representative images of whole larvae infected with LD of the Wasabi-expressing *M. marinum* ΔRD1 mutant, at 48 hpA (Scale bar = 500 µm). **(B)** Graph represents the FPC after infection with LD of ΔRD1 at 96 hpi, corresponding to 48 hpA. **(C)** Fin length after injection of PBS (CT) or infection with LD of ΔRD1 at 48 hpA (mean ± SEM, n< 30, Mann Whitney test, two-tailed, non-significant). **(D)** Fin area after injection of PBS (CT) or infection with LD of ΔRD1 at 48 hpA (mean ± SEM, n< 30, Mann Whitney test, two-tailed, non-significant). **(E)** Representative images of whole larvae infected with LD, MD and HD of the Wasabi-expressing *M. marinum* ΔRD1 mutant, at 72 hpA (Scale bar=500 µm). **(F)** Graph represents the FPC after infection with LD, MD and HD of ΔRD1 at 120 hpi, corresponding to 72 hpA (mean ± SEM, n<30, Kruskal-Wallis, Dunn’s multiple comparisons test, **p<0.01, ****p<0.0001). **(G)** Fin length after injection of PBS (CT) or infection with LD, MD and HD of ΔRD1 at 72 hpA (mean ± SEM, n>30, ordinary one-way ANOVA, Dunnett’s multiple comparisons test, non-significant). **(H)** Fin area after injection of PBS (CT) or infection with LD, MD and HD of ΔRD1 at 72 hpA (mean ± SEM, n>30, ordinary one-way ANOVA, Dunnett’s multiple comparisons test, non-significant). **(I)** Wound contour straightness after injection of PBS (CT) or infection with LD, MD and HD of ΔRD1 at 72 hpA (mean ± SEM, n>30, ordinary one-way ANOVA, Dunnett’s multiple comparisons test, non-significant).

Supplementary Figure 3 Infection influence cell proliferation and mesenchymal cell morphology **(A)** *Tg(rcn3:GAL4/UAS:mCherry)* larvae were either kept intact (uncut), amputated (cut) following LD infection with *M. marinum*. Confocal Z projections of caudal fin with mCherry-F fluorescence were imaged at 48 hpA (Scale bar = 60 µm). **(B)** *Tg(rcn3:GAL4/UAS:mCherry)* larvae were either kept intact (uncut), amputated (cut) following MD and HD infection with *M. marinum*. Confocal Z projections of caudal fin with mCherry-F fluorescence were imaged at 72 hpA (Scale bar=60 µm). **(C)** Representative images of total macrophages (red) in whole larvae after injection of PBS (CT) or infection with LD, MD and HD of *M. marinum* without amputation at 72 hpf and at 1 hpA (Scale bar = 100 µm).

Supplementary Figure 4
*M. marinum* infection impacts recruitment and activation of macrophages in the regenerated caudal fin and leads to granulomas formation. **(A)** Quantification of the number of *mpeg* ^+^ cells in the fin tip at 1 hpA after injection of PBS (CT) or infection with LD, MD and HD of *M. marinum* ΔRD1 mutant (mean ± SEM, n<30, Kruskal-Wallis, Dunn’s multiple comparisons test, non-significant). **(B)** Quantification of the number of *mpeg* ^+^-positive cells in the entire fin at 1 hpA after injection of PBS (CT) or infection with LD, MD and HD of ΔRD1 (mean ± SEM, n<30, Kruskal-Wallis, Dunn’s multiple comparisons test, non-significant). **(C)** Representative images of aggregated macrophages (red) and bacteria (green, MD infection) at 48 hpA, highlighting the presence of granulomas (left panels) (Scale bar=100 µm). Number of granulomas at 48 hpA after injection of LD, MD and HD of *M. marinum* (right panel) (mean ± SEM, n< 30, Kruskal-Wallis, Dunn’s multiple comparisons test, ***p ≤ 0.001, ****p ≤ 0.0001). **(D)** Representative images of total macrophages (red) in the whole larva after injection of PBS (CT) or infection with MD and HD of *M. marinum* at 72 hpA (Scale bar=500 µm **(E)** Representative images of total macrophages (red) in the whole larva after infection of an HD of *M. marinum* at 72 hpA, in the *Tg(mpeg1:mCherry-F) versus Tg(mfap4:mCherry-F)* transgenic lines (Scale bar = 500 µm). **(F)** Representative images of total macrophages (red) in the tail after infection of a HD of *M. marinum* at 72 hpA in the *Tg(mpeg1:mCherry-F) versus Tg(mfap4:mCherry-F)* transgenic lines (Scale bar = 100 µm). **(G)** Quantification of the number of *mpeg* ^+^ cells in the entire fin at 48 hpA after infection with a HD of *M. marinum* in the *Tg(mpeg1:mCherry-F) versus Tg(mfap4:mCherry-F)* transgenic lines (mean ± SEM, n< 30, Mann Whitney test, two-tailed, non-significant). **(H)** Quantification of the number of *mpeg* ^+^ cells in the entire fin at 72 hpA after infection with a HD of *M. marinum* in the *Tg(mpeg1:mCherry-F) versus Tg(mfap4:mCherry-F)* transgenic lines (mean ± SEM, n<30, Mann Whitney test, two-tailed, non-significant).

Supplementary Figure 5
*M. marinum* infection impacts on cell viability. **(A)** Number of total macrophages *mcherry* ^+^ in the whole larva after injection of PBS (CT) or infection with LD, MD or HD of *M. marinum* at 48 hpA, determined by flow cytometry (mean ± SEM, n = 4-5, Mann Whitney test, two tailed, non-significant). **(B)** Dot plot representing the Annexin V and 7AAD staining, performed using the *mcherry* ^+^ cells isolated from *Tg(mpeg1: mCherry-F)* larvae at 48 hpA after injection of PBS (CT) or infection with LD, MD or HD of *M. marinum* and amputation.

Movie 1 Infection influences collagen fibers in the regenerated caudal fin. **(A)** Times lapses and second harmonic Z projections were done after injection of PBS (CT) at 72 hpA (Scale bar = 60µm).

Movie 2 Infection influences collagen fibers in the regenerated caudal fin. **(A)** Time lapse and second harmonic Z projections were done after injection of LD of *M.marinum* at 72 hpA (Scale bar = 60µm).

Movie 3 Infection influences collagen fibers in the regenerated caudal fin. **(A)** Time lapse and second harmonic Z projections were done after injection of MD of *M. marinum* at 72 hpA (Scale bar = 60µm).

Movie 4 Infection influences collagen fibers in the regenerated caudal fin. **(A)** Time lapse and second harmonic Z projections were done after injection of HD of *M. marinum* at 72 hpA (Scale bar = 60µm).

Movie 5 Dynamic of macrophages mobilization at the caudal fin after amputation. **(A)** Time lapse and Z projections were done with confocal microscopy after amputation in a non-infectious condition of the *Tg(mpeg1: mCherry-F)*: 15 min pA to 12 hpA corresponding of the arrival of the macrophages line at the fin tip (Scale bar = 60 µm).

Movie 6 Dynamic of macrophages mobilization at the caudal fin after amputation. **(A)** Time lapse and Z projections were done with confocal microscopy after amputation in a non-infectious condition of the *Tg(mpeg1: mCherry-F)*: 5 to 17 hpA corresponding of the maintain of the macrophages line at the fin tip (Scale bar = 60 µm).

Movie 7 Dynamic of macrophages mobilization at the caudal fin after amputation. **(A)** Time lapse and Z projections were done with confocal microscopy after amputation in a non-infectious condition of the *Tg(mpeg1: mCherry-F)*: 18 to 30 hpA corresponding of the departure of the macrophages line at the fin tip (Scale bar = 60 µm).
