# Supplementary material for: Exploring Macrophage-Dependent Wound Regeneration During Mycobacterial Infection in Zebrafish
Source: Front Immunol. 2022 Mar 24;13:838425. doi: 10.3389/fimmu.2022.838425 (PMC8987025; doi:10.3389/fimmu.2022.838425)

Supplementary figure 1.

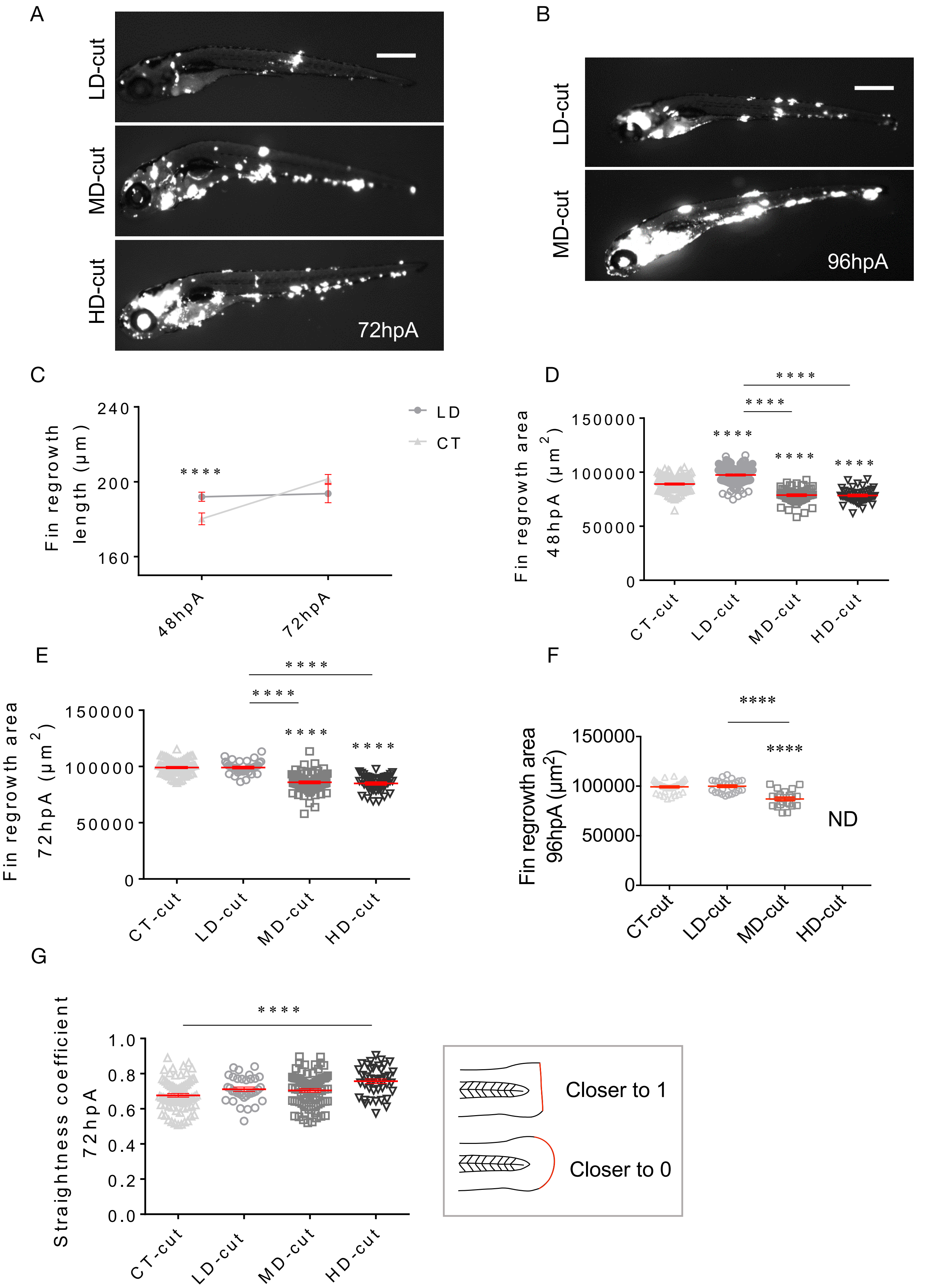

Supplementary figure 2.

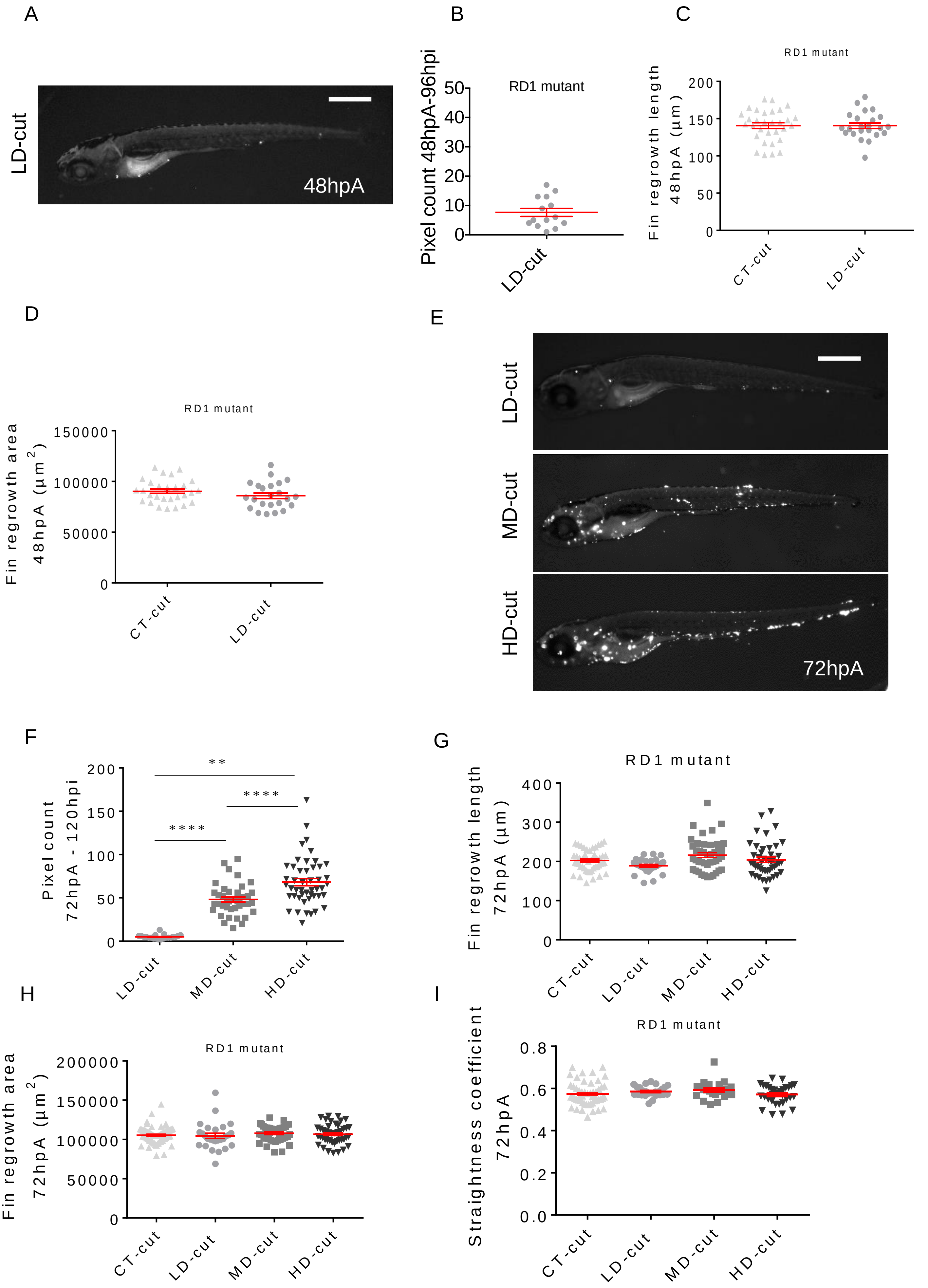

Supplementary figure 3.

A

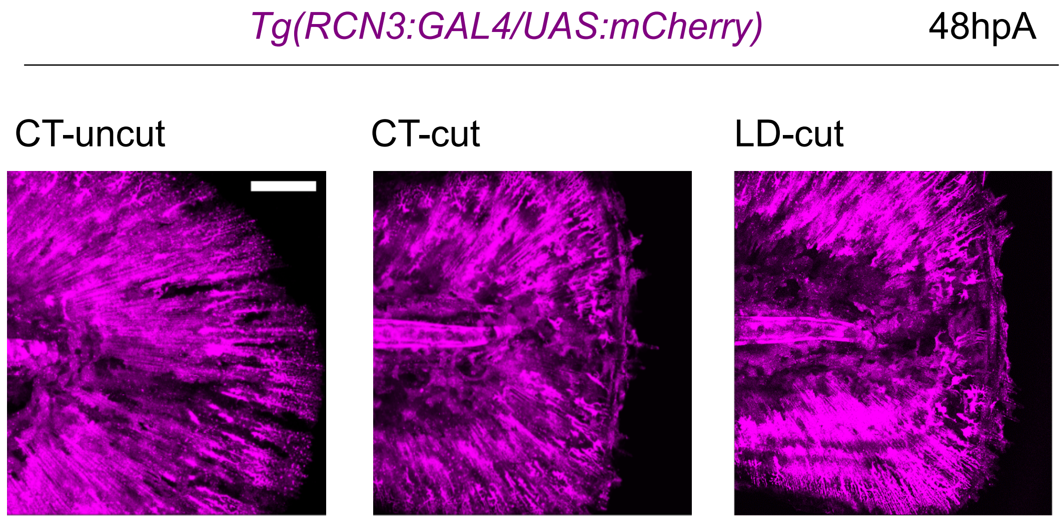

B

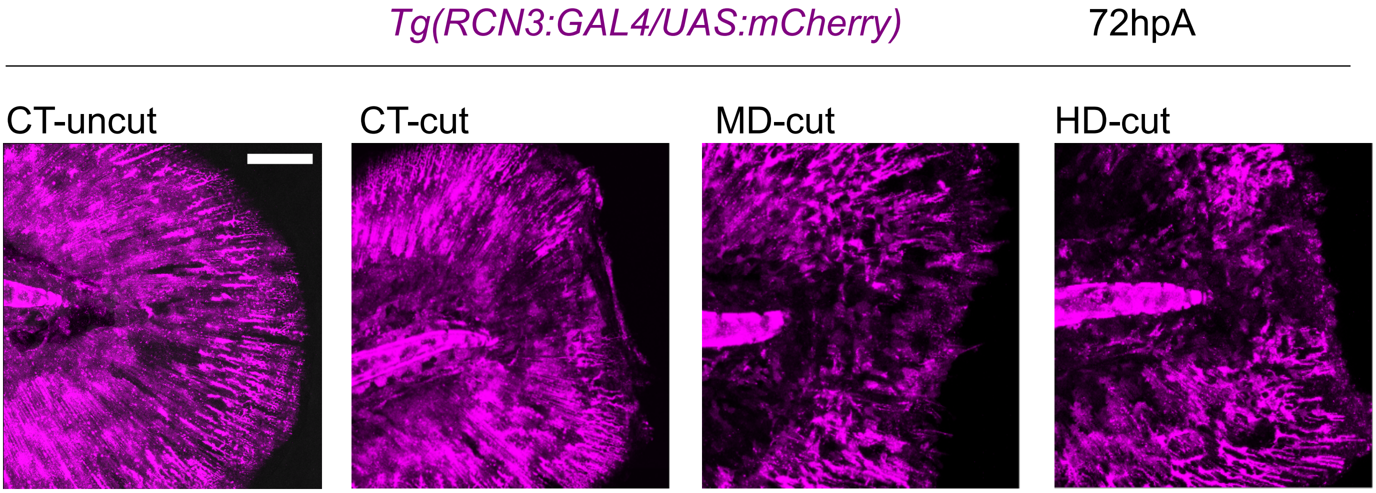

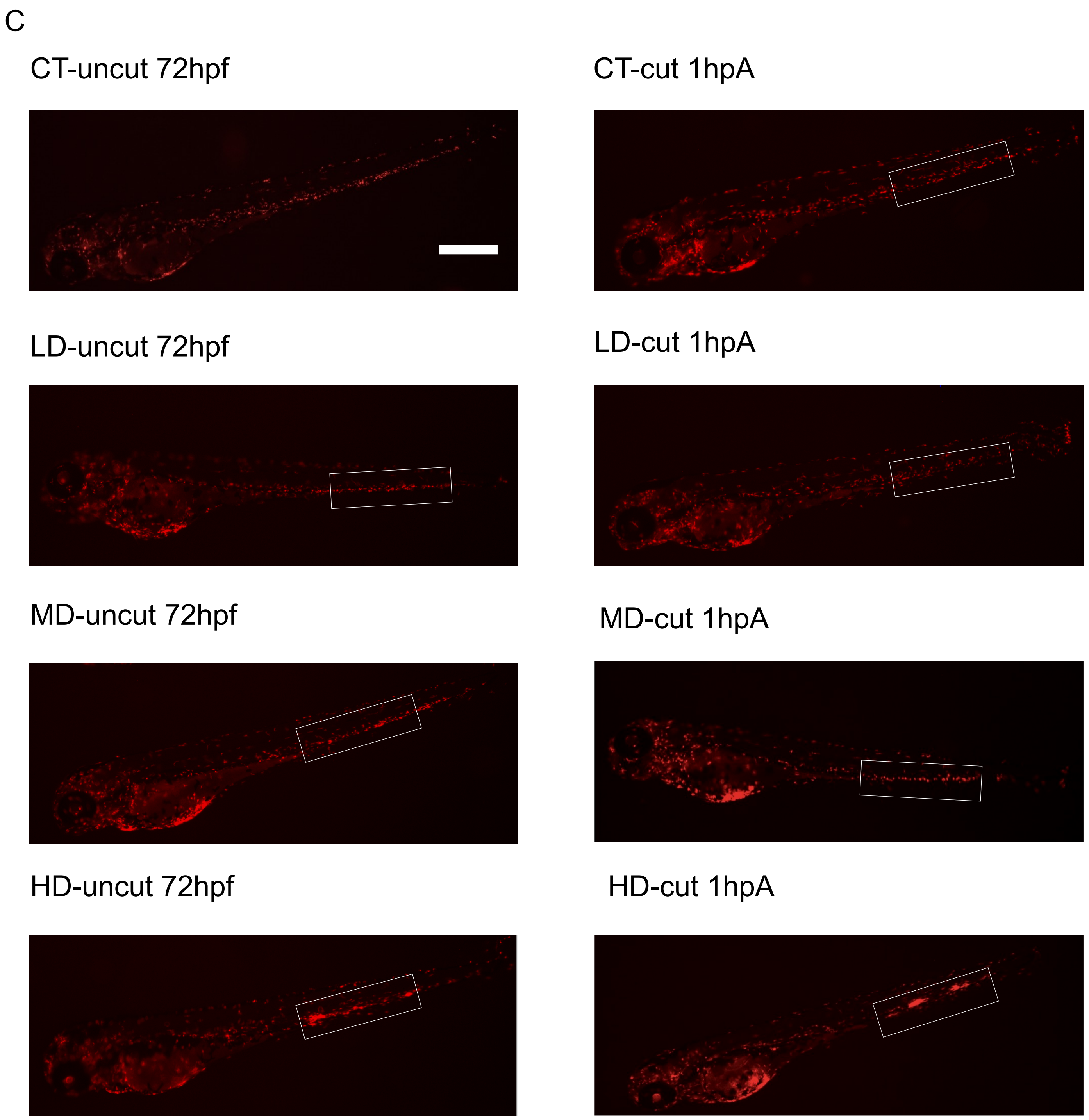
C
CT-uncut 72hpf
CT-cut 1hpA
LD-uncut 72hpf
LD-cut 1hpA
MD-uncut 72hpf
MD-cut 1hpA
HD-uncut 72hpf
HD-cut 1hpA

Supplementary figure 4.

A

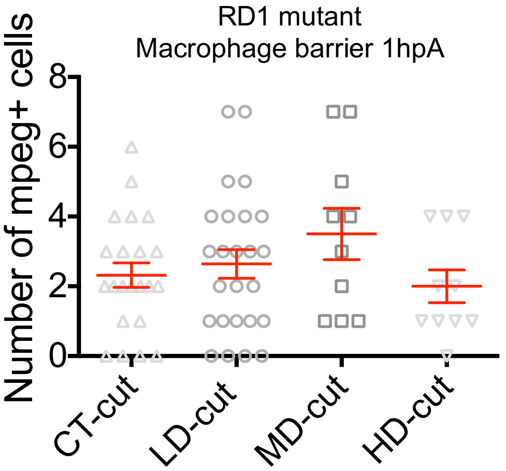

B

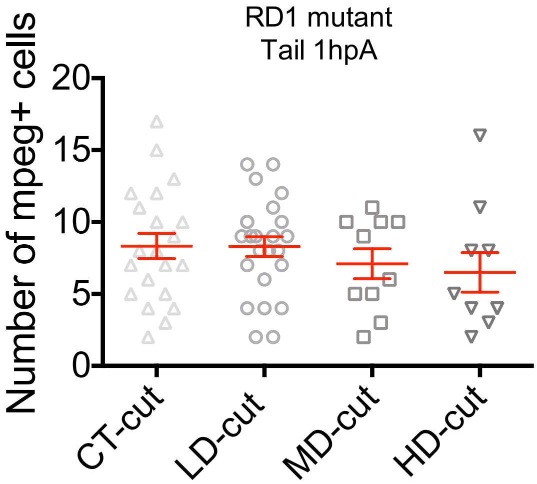

C

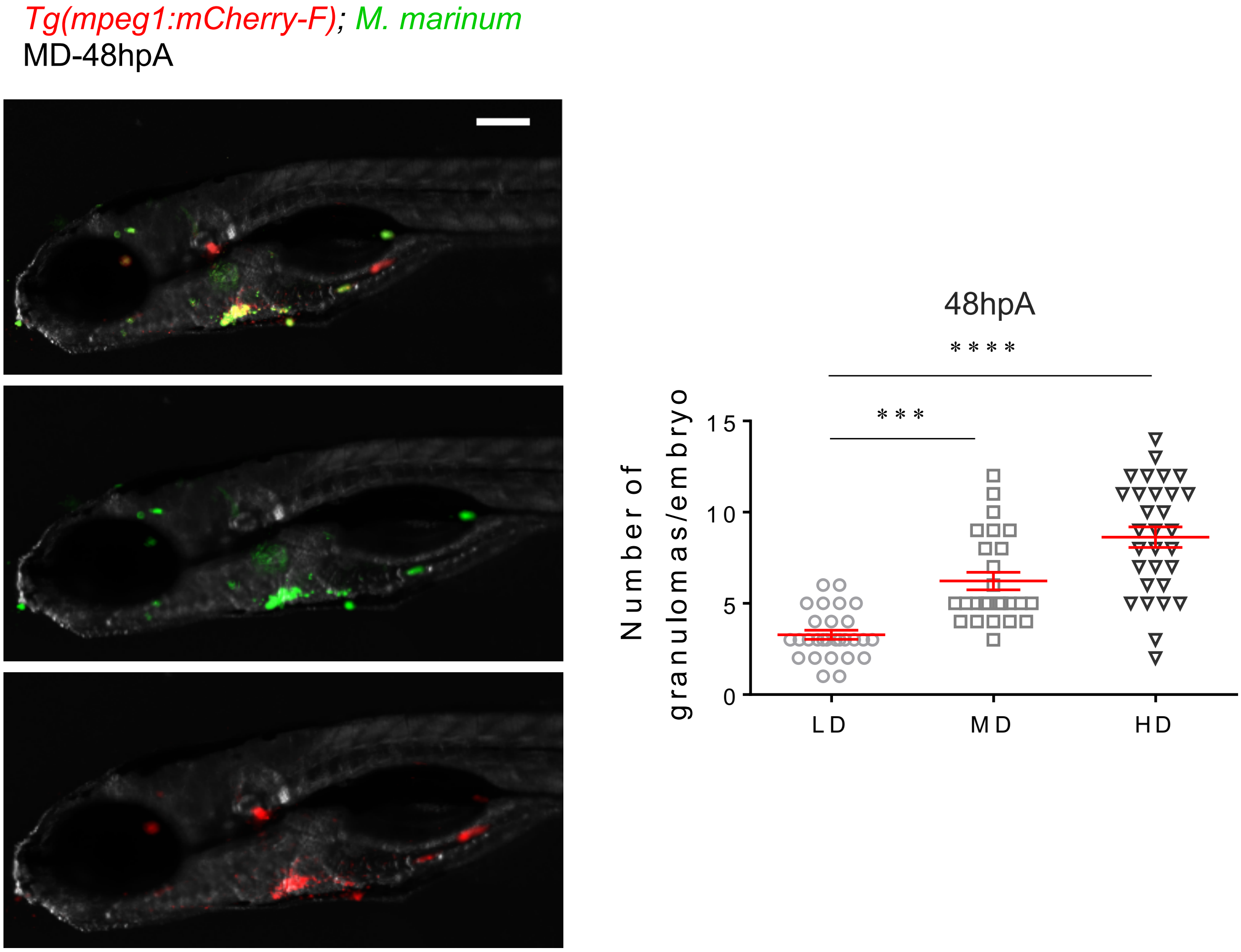

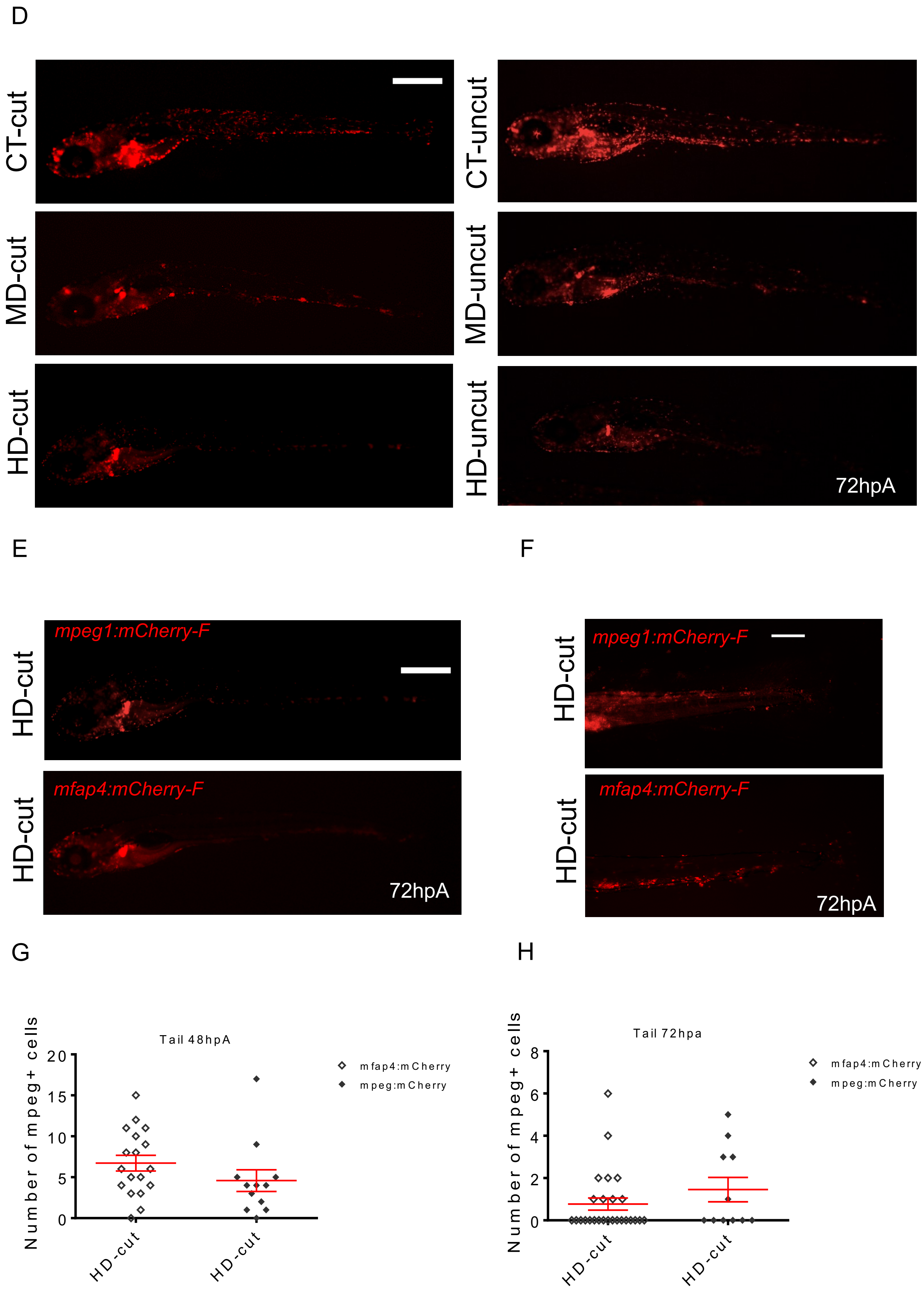

D
CT-cut
CT-uncut
MD-cut
MD-uncut
HD-cut
HD-uncut
72hpA
E
F
HD-cut
mpeg1:mCherry-F
HD-cut
mfap4:mCherry-F
72hpA
HD-cut
mpeg1:mCherry-F
HD-cut
mfap4:mCherry-F
72hpA
G
H
Tail 48hpA
Number of mpeg+ cells
0
5
10
15
20
mfap4:mCherry
mpeg:mCherry
HD-cut
HD-cut
Tail 72hpa
Number of mpeg+ cells
0
2
4
6
8
mfap4:mCherry
mpeg:mCherry
HD-cut
HD-cut

Supplementary figure 5.

A

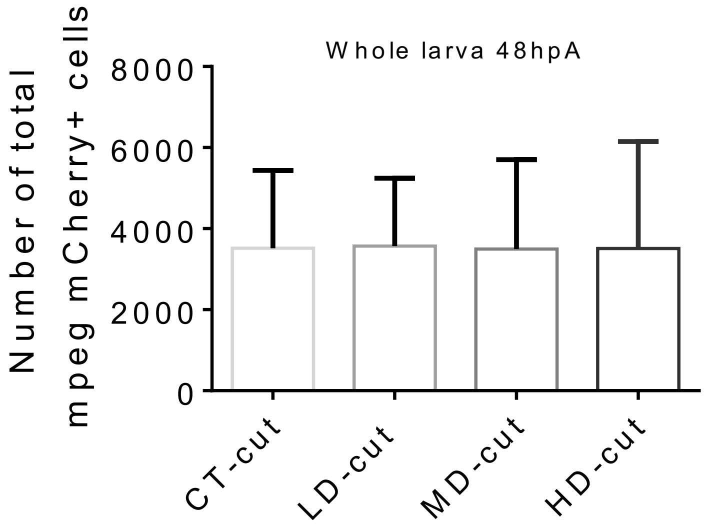

B

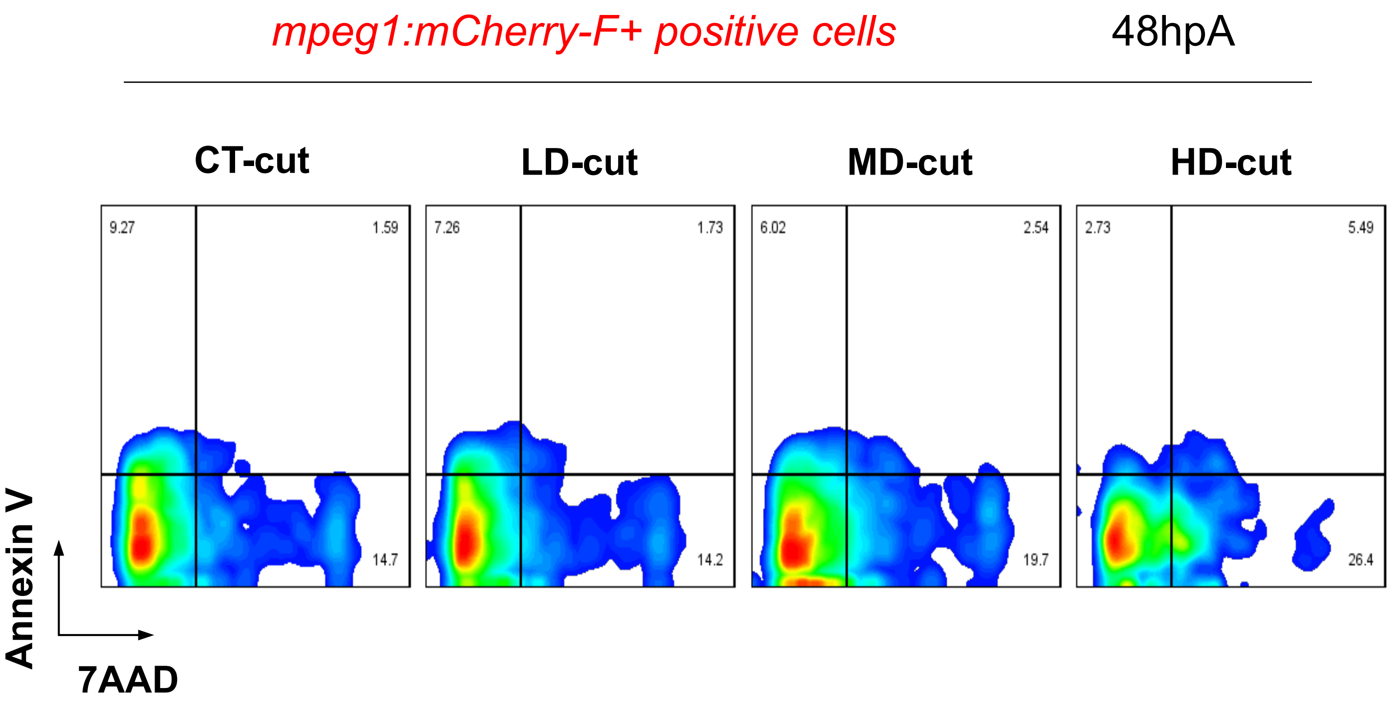

Supplement: Supplementary Figure 1 — Caudal fin regrowth is conditioned by the severity of the infection. (A) Representative images of whole larvae infected with LD, MD or HD of M. marinum expressing Wasabi, at 72 hpA (Scale bar = 500 µm). (B) Representative images of whole larvae infected with LD, MD or HD of M. marinum expressing Wasabi, at 96 hpA (Scale bar = 500 µm). All larvae died with the HD inoculum at 96 hpA. (C) Graph represents the kinetic of fin length after injection of PBS (CT) or LD of M. marinum, between 48 and 72 hpA (mean ± SEM, n>30, t-test, two-tailed, **** p<0.0001). (D) Graphs showing the fin area after injection of PBS (CT) or infection with LD, MD or HD of M. marinum at 48 hpA (mean ± SEM, n> 30, ordinary one-way ANOVA, Dunnett’s multiple comparisons test, compared to control except when indicated, ****p ≤ 0.0001). (E) Graphs showing the fin area after injection of PBS (CT) or infection with LD, MD or HD of M. marinum at 72 hpA (mean ± SEM, n>30, ordinary one-way ANOVA, Dunnett’s multiple comparisons test, compared to control except when indicated, ****p ≤ 0.0001). (F) Graphs showing the fin area after injection of PBS (CT) or infection with LD, MD or HD of M. marinum at 96 hpA (mean ± SEM, n<30, Kruskal-Wallis, Dunn’s multiple comparisons test, compared to control except when indicated, ****p ≤ 0.0001). All larvae died with the HD infection at this timepoint. ND, not determined. (G) Graphs represent the wound contour straightness of the fin after injection of PBS (CT) or LD, MD and HD of M. marinum, at 72 hpA (mean ± SEM, n>30, ordinary one-way ANOVA, Dunnett’s multiple comparisons test, ****p≤ 0.0001). Explanatory diagram. [file DataSheet_1.pdf]
